# Microdialysis-Assessed Exercised Muscle Reveals Localized and Differential IGFBP Responses to Unilateral Stretch Shortening Cycle Exercise

**DOI:** 10.3389/fendo.2020.00315

**Published:** 2020-05-29

**Authors:** Bradley C. Nindl, Juha Ahtiainen, Sheila S. Gagnon, Ritva S. Taipale, Joseph R. Pierce, Brian J. Martin, Meaghan E. Beckner, M. Lehti, Keijo Häkkinen, Heikki Kyröläinen

**Affiliations:** ^1^Neuromuscular Research Laboratory/Warrior Human Performance Research Center, University of Pittsburgh, Pittsburgh, PA, United States; ^2^US Army Research Institute of Environmental Medicine, Natick, MA, United States; ^3^Army Public Health Center, Aberdeen Proving Ground, MD, United States; ^4^Neuromuscular Research Center, Faculty of Sport and Health Sciences, University of Jyväskylä, Jyvaskyla, Finland

**Keywords:** microdialysis, IGF-I, stretch shortening cycle exercise, interstitial fluid, binding proteins, muscle

## Abstract

Microdialysis allows for a preview into local muscle metabolism and can provide physiological insight that blood measurements cannot.

**Purpose:** To examine the potential differential IGF-I system regulation in interstitial fluid during unilateral stretch shortening cycle exercise.

**Methods:** 10 men (26 ± 7 year) performed unilateral jumping [stretch shortening cycle (SSC) exercise at 50% of optimal jump height] until volitional fatigue on a sled apparatus. Biological sampling took place using a catheter inserted into an antecubital vein (serum), and 100 kDa microdialysis probes inserted into the thigh muscle of each exercise/control leg (dialysate). Serum was drawn before (Pre; −3 h) and after SSC [Post I (+0 h), II (+3 h), or III (+20 h)]; dialysate was sampled for 2 h before (Pre), during/immediately after (Ex), and 3 h into recovery (Rec) following SSC. IGF-I system parameters (free/total IGF-I and IGFBPs 1–6) were measured with immunoassays. Interstitial free IGF-I was estimated from dialysate IGF-I and relative recovery (ethanol) correction. Data were analyzed with repeated measures ANOVA.

**Results:** Serum total IGF-I remained elevated +3 h (Post II: 182.8 ± 37.6 vs. Pre: 168.3 ± 35.0 ng/mL, *p* < 0.01), but returned to baseline by +20 h (Post III vs. Pre, *p* = 0.31). No changes in serum free IGF-I were noted. Serum BP-1 and −3 increased over baseline, but not until + 20 h after SSC (Post III vs. Pre: 7.6 ± 4.9 vs. 3.7 ± 2.3 and 1,048.6 ± 269.2 vs. 891.4 ± 171.2 ng/mL, respectively). We observed a decreased serum BP-6 +3 h after SSC (*p* < 0.01), followed by a return to baseline at +20 h (*p* = 0.64 vs. Pre). There were no exercise-induced changes in serum BP-2, −4, or −5. Unlike serum, there were no changes in dialysate or interstitial free IGF-I in either leg (*p* > 0.05). Dialysate BP-1 remained increased in both exercise and control legs through 3 h into recovery (Rec vs. Pre, *p* < 0.01). Dialysate BP-3 also demonstrated a prolonged elevation over Pre SSC concentrations, but in the exercise leg only (Ex and Rec vs. Pre, *p* < 0.04). We observed a prolonged decrease in dialysate BP-5 (Ex and Rec vs. Pre, *p* < 0.03) and an increase in BP-4 IP in the exercise leg only. There were no changes relative to Pre SSC in dialysate BP-2 or −6.

**Conclusions**: Unilateral exercise drives differential regulation of the IGF-I system at both local and systemic levels. More specifically, this is the first study to demonstrate that localized exercise increases IGFBP-3, IGFBP-4 and decreases in IGFBP-5 in muscle interstitial fluid.

## Introduction

The insulin-like growth factor-I (IGF-I) system serves as an important metabolic modulator for physiological processes related to growth, development, muscle repair/regeneration and altered energy and activity paradigms (1–8). The IGF-I system circulates and is present across a number of different biocompartments (i.e., blood, interstitial fluid, and muscle) (9). The IGF-I system is comprised of IGF-I itself and a family of six different binding proteins (BPs 1–6) (1, 10–12). The biological action IGF-I is influenced by a family of BPs that can serve to either stimulate or inhibit IGF-I action (1, 10–12). A recent special edition in Frontiers in Endocrinology highlights the important role of IGFBPs for increasing functional diversity and utility for discerning context-dependent roles for IGF-I action and signaling (3, 10). While IGFBPs are known to serve as carrier, sequestering, and trafficking reservoirs both potentiating and inhibiting IGF-I action, they have also been demonstrated to possess IGF-independent actions (1, 10–12). We have previously observed that acute resistance exercise had no impact on overnight IGF-I concentrations, but alterations were detected for IGFBPs suggesting that exercise could influence the manner in which IGF-I is partitioned among its family of BPs (13). More recently, our laboratory has further reported the importance of measuring IGFBPs by demonstrating that 8 weeks of exercise training resulted in increased basal IGFBPs 2 and 3, but no change in total IGF-I and have recommended moving beyond solely relying on measures of total IGF-I concentrations for insight into IGF-I physiological action (14).

The modulatory influence of IGFBPs on IGF-I action is well established across various *in vitro* tissues and cell types, but much less studied and understood within the context of exercise responses and adaptations (2, 7, 15). Awede et al. (16) were the first to demonstrate the influence of loading on IGFB-4 and IGFBP-5 gene expression using a murine model, illustrating the potentially critical mechanistic role for IGFBPs mechano-transduction in muscle adaptation. Further, most exercise studies have measured the IGF-I system within the systemic circulation. IGF-I activity in interstitial fluid most proximal to tissues and cells could provide potentially meaningful context to understanding how exercise conveys hormonal and biochemical signals (4, 7, 9, 15, 17–19). Microdialysis is a method to sample interstitial fluid allowing for a preview into local muscle metabolism, and can provide physiological insight that blood measurements cannot (9, 17, 20–23). For example, we have previously demonstrated that post-exercise IGF-I increases in the systemic circulation are not reflected in post-exercise interstitial fluid (ISF) (9). To date, we are only aware of two studies measuring IGFBPs in ISF following exercise (21, 24). Berg et al. (21) reported no change in IGFBP-1 and IGFBP-3 proteolysis in exercised muscle via microdialysis and Olesen et al. (24) reported that IGFBP-4 was increased in peritendinous tissue after running exercise, suggesting a key role in human collagen synthesis.

As both IGF-I and IGFBPs are produced systemically and locally, it has proven difficult to discern the relative impact of exercise-mediated whole-body metabolic-stress (i.e., systemic) vs. muscle/load specific (i.e., local) on subsequent IGF-I system responses (2, 7, 15). Also of particular note, stretch-shortening cycle (SSC) exercise is a unique model to study the interaction between neural, mechanical, structural and biochemical events (25, 26). An advantage of SSC over isolated concentric and eccentric contractions is that SSC exercise more closely mimics the loading of the neuromuscular system observed in normal human locomotion (26). To more fully examine the IGF-I system (both IGF and IGFBPs) across biocompartments in exercised muscle, we sought to determine whether IGFBPs were differentially influenced by SSC exercise using microdialysis to sample ISF from both an exercised limb vs. a control limb with simultaneous blood sampling.

## Methods

### Subjects

Ten healthy men (age: 26 ± 7 year; height: 180 ± 8 cm, body mass: 77.4 ± 10.7 kg) volunteered after being briefed on all study methods, risks, and discomforts, and after providing their verbal and written consent. Participants' height was measured to the nearest 0.1 cm using a stadiometer and body mass was measured using a standard electronic scale to the nearest 0.1 kg. The study protocol was performed according to the Declaration of Helsinki, and was approved by the Commission on Ethics of the University of Jyväskylä prior to implementation.

### Experimental Design Overview

Subjects were asked to refrain from caffeine, alcohol, and exercise for 24 h prior to the experimental visit. On the morning of the visit, overnight-fasted subjects had a venous catheter inserted into their antecubital vein for systemic blood draws, and microdialysis probes inserted into their thigh (vastus lateralis; VL) muscle to sample local skeletal muscle ISF (dialysate). Using sterile procedures, microdialysis probes were inserted into the VL of each leg, which has been described elsewhere in detail (9). Briefly, a small amount (~2 cc per insertion site) of 1% lidocaine was injected just under the skin above the distal VL on both legs. Next, two pre-sterilized non-linear 100 kDa molecular weight cut-off probes (30 mm membrane; MDialysis Inc., N. Chelmsford, MA) were placed in the VL muscle of each leg using an 18-gauge needle and manufacturer provided removable sheath introducer on each leg. The introducer was removed, leaving only the probe membrane embedded in the muscle. Once all probes were inserted and secured to the leg, the inlet tubing was connected to a portable CMA adjustable flow rate pump (CMA-107; set to 2 μL/min) containing a syringe filled with sterile perfusate solution [0.9 % sodium chloride, 30 g/L Dextran 40 (Pharmacosmos, Holbaek, Denmark), and 10 mM ethanol (EtOH)]. Dextran was added to prevent fluid loss across the large pore size membrane (ultrafiltration), and EtOH was added to qualitatively estimate changes in microvascular blood flow and to calculate interstitial concentrations. After a 5 min flush sequence, perfused probes were left in tissue for at least 45–60 min prior to any sampling. Due to the extended time period of the experimental visit, subjects were provided a small food bar (180 kcal) before and after the exercise protocol.

### Acute Exercise Protocol

In order to study the localized effects in an exercise vs. control muscle, subjects performed a unilateral jumping exercise utilizing the stretch shortening cycle (SSC) following a standardized 0 min warm-up on a bicycle ergometer 60–70% of maximum HR. Utilizing a specially designed sled apparatus described previously (26), subjects performed unilateral jumps with the dominant leg, keeping the contralateral leg isolated/passive during exercise. Thus, we were able to have the one leg serve as an internal control when comparing local muscle IGF-I responses to exercise. Subjects were secured on the sled apparatus using straps to prevent unnecessary movement during the jumping motions. The unilateral jumps began from a knee angle set to 107° from flexion (measured via a goniometer) with the sled set at 23° from the horizontal plane. The jumping protocol was set at 50% of the individual optimal jump (optimal parameters were determined in a separate assessment) and continued until volitional fatigue. For the separate assessment, optimal drop heights were determined individually for each subject by having them dropped on the sledge-jump from different heights to determine their rising height (25, 26).

The number of fatiguing jumps performed ranged from 60 to 1,280.

### Biological Sampling

**Figure 1:**
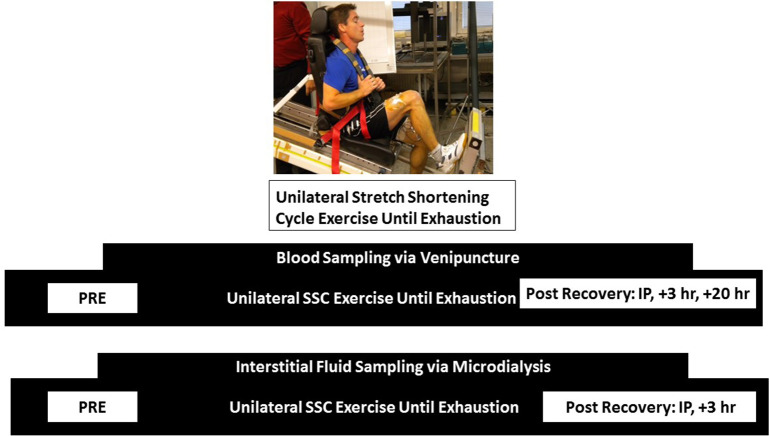
Sampling schematic for blood via venipuncture and interstitial fluid via microdialysis. Samples were obtained at the same timepoints for pre, immediate post, and +3 h post timepoints. An additional blood sample was obtained at +20 h post.

At pre-determined time points before (Pre; −3 h before exercise) and after (Immediately Post/Post I: 0 h; Post II: +3 h; Post III: +20 h) the SSC exercise, subjects had blood drawn from the venous catheter. Serum was separated after allowing the blood sample to clot at room temp for 30 min and after centrifugation at 3,000 g for 15 min. Skeletal muscle dialysate samples were collected 2-h prior to (Pre), immediately following exercise (Ex); including time during the SSC exercise), and 3 h post-exercise recovery (Rec). Sample vials were weighed prior to and following each collection in order to monitor flow rate. Dialysate was run immediately for EtOH, and the remaining aliquot was stored at −20°C until specific IGF-I system analysis occurred. Figure 1 depicts the sampling timelines for blood and interstitial fluid.

### Assays

Serum and dialysate samples were analyzed for several IGF-I system components, and all samples for a particular analyte were run in the same assay batch to minimize intra-assay variance. Serum Total IGF-I was analyzed on the Immulite 1000 (Siemens Healthcare Diagnostics, Malvern, PA; LKGF1 kit, reported sensitivity of 20 ng/mL). Serum and dialysate Free IGF-I was analyzed with an ELISA from Beckman Coulter (Brea, CA; DSL-10-9400 kit, reported sensitivity of 0.015 ng/mL), and quantified on a Dynex MRX Revelation absorbance reader (Dynex Technologies, Chantilly, VA). Serum and dialysate IGFBP-1 through BP-6 were analyzed on a multiplexed bead-based fluorescent assay from Millipore (Billerica, MA; HIGFBP-53K multiplex kit, reported sensitivity of 0.013, 0.325, 0.145, 0.573, 1.15, and 0.078 ng/mL, respectively), and quantified on the Luminex 200 Instrument. Intra-assay CVs for the respective assays were as follows: Total IGF-I = 4.4%; Free IGF-I = 8.7%; BP-1 through BP-6 ranged 6.2–12.5%.

Perfusate and dialysate samples were run for EtOH concentrations using a clinical analyzer. A subsequent calculation of the outflow: inflow ratio (O:I) from [dialysate EtOH]/[perfusate EtOH], allowed a qualitative estimate of local microvascular blood flow (where O:I is inversely proportional to local blood flow), and subsequent estimation of interstitial analyte concentrations from measured dialysate analyte concentrations since EtOH is not metabolized by the local tissue. Thus, changes in EtOH concentration reflect changes in blood flow.

### Interstitial Estimates

As the interstitial concentration of an analyte will depend on changes in production and clearance rates, the changes in microvascular blood flow to a tissue (e.g., changes in EtOH O:I ratio) can be used to estimate/calculate interstitial concentrations from assayed dialysate concentrations. Using the external standard approach and separate *in vitro* experiments to determine the relative recovery rates of analyte to EtOH under various conditions, we calculated the *in vivo* interstitial concentration of multiple IGF-I system components (free IGF-I, IGFBPs 1-6) using the following equation: [*in vivo* interstitial analyte] = [(*in vitro* EtOH relative recovery/*in vitro* analyte relative recovery) × (*in vivo* dialysate analyte)]/[1–(*in vivo* EtOH O:I)]. This interstitial estimation is similar to interstitial corrections previously reported (23, 27).

### Statistical Analyses

All data are presented as mean ± standard deviation, unless otherwise noted. Acute changes in serum IGF-I system components were assessed with repeated measures ANOVA (RMANOVA), using within-subjects time factor (Pre, Post I, II, III). Acute changes in dialysate/interstitial IGF-I system components were assessed with a RMANOVA, using within-subjects time factor (Pre, Ex, Rec) and were analyzed in each leg (exercise, control) separately. If the RMANOVA model detected a significant F-ratio (*p* < 0.05), *post-hoc* comparisons were tested with an LSD test. All analyses were conducted using SPSS Statistics v. 21 (IBM, Artmonk, NY).

## Results

### Serum Total and Free IGF-I

As a result of unilateral SSC exercise, there was an acute increase in serum total IGF-I at the Post I and Post II time points (*p* < 0.01 for both vs. Pre), and by Post III, serum total IGF-I had returned to baseline values (*p* = 0.31 vs. Pre). In contrast, there were no acute changes in serum free IGF-I with the SSC exercise (*p* = 0.55) (Refer to Figure 1).

### Serum IGF-I Binding Proteins 1-6

**Figure 2:**
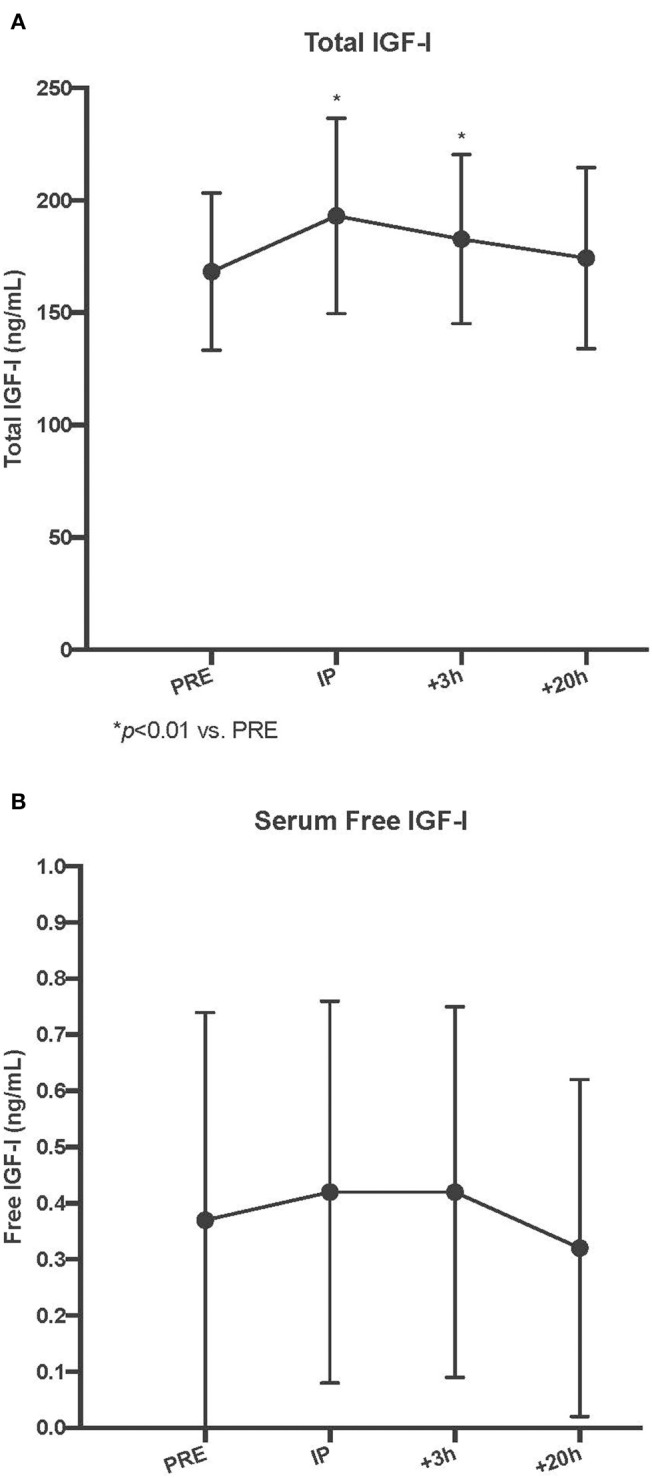
Circulating serum total **(A)** and free insulin-like growth factor-I (IGF) **(B)** concentrations pre, immediately post (IP), 3 h post +3 h, and 20 h post (+20 h) to unilateral stretch shortening exercise. *denotes statistical difference from pre-exercise (*p* < 0.05).

BP-1 demonstrated a delayed increase in serum concentrations but not until the +20 h time point (different from all preceding time points, *p* < 0.01). There were no changes observed with BP-2 (*p* = 0.52). As with BP-1, there was an increase in serum BP-3, but not until the +20 h time point, which was the only significant difference from baseline (*p* = 0.05 vs. Pre). We observed a main time effect for serum BP-4 (*p* < 0.01); however, *post-hoc* testing revealed that the apparent increase at the immediate post time point did not reach statistical significance (*p* = 0.07 vs. Pre). There were significant reductions in the circulating BP-4 pool during the recovery time period (Post II and Post III vs. Post I, *p* < 0.05). There was no acute change observed for serum BP-5 (*p* = 0.06). In a pattern different from all other serum BP responses, we observed a significant decrease in serum BP-6 at the + 3 h post time point (*p* < 0.02 vs. all other time points) (Refer to Figure 2).

### Dialysate Free IGF-I

**Figure 3:**
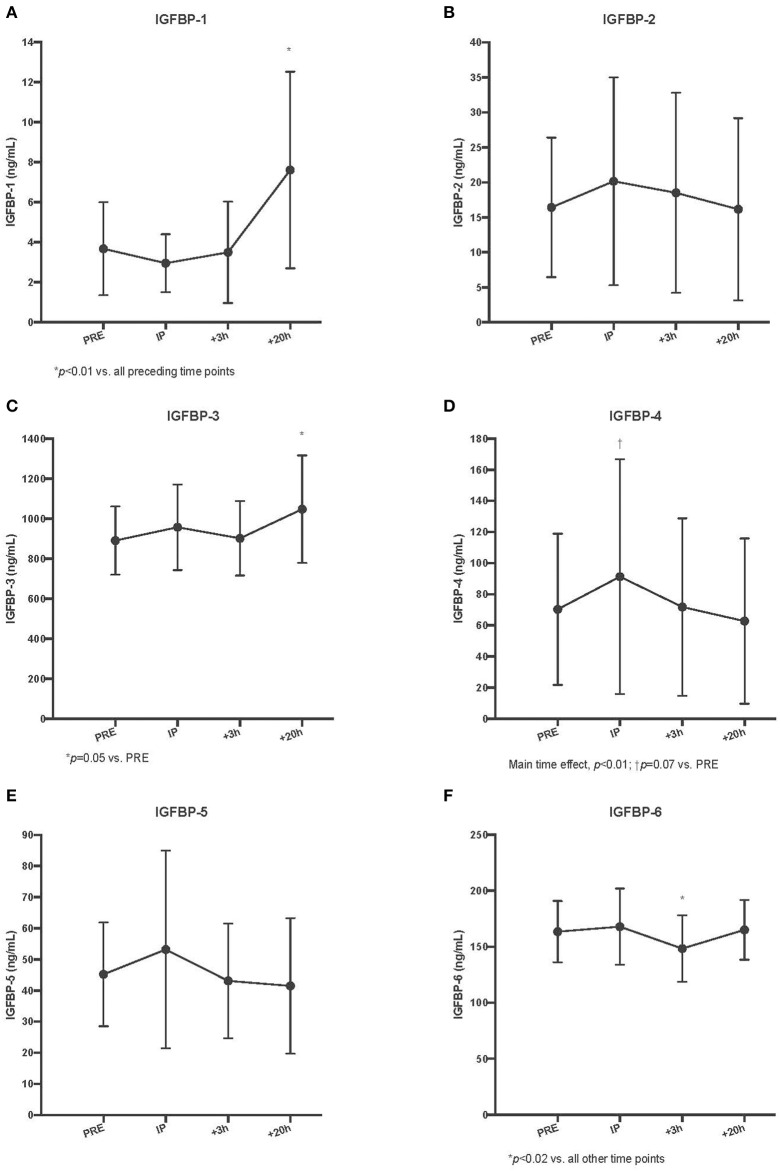
Circulating insulin-like growth factor-I binding protein (IGFBP) concentrations for IGFBP-1 **(A)**, IGFBP-2 **(B)**, IGFBP-3 **(C)**, IGFBP-4 **(D)**, IGFBP-5 **(E)**, and IGFBP-6 **(F)** pre, immediately post (IP), 3 h post (+3 h), and 20 h post (+20 h) to unilateral stretch shortening exercise. *denotes statistical difference from pre-exercise (*p* < 0.05).

As with serum free IGF-I, there were no acute changes with dialysate free IGF-I following SSC exercise, in either the exercise (*p* = 0.62) or control (*p* = 0.70) leg musculature (Refer to Figure 3).

### Dialysate IGF-I Binding Proteins 1-6

**Figure 4:**
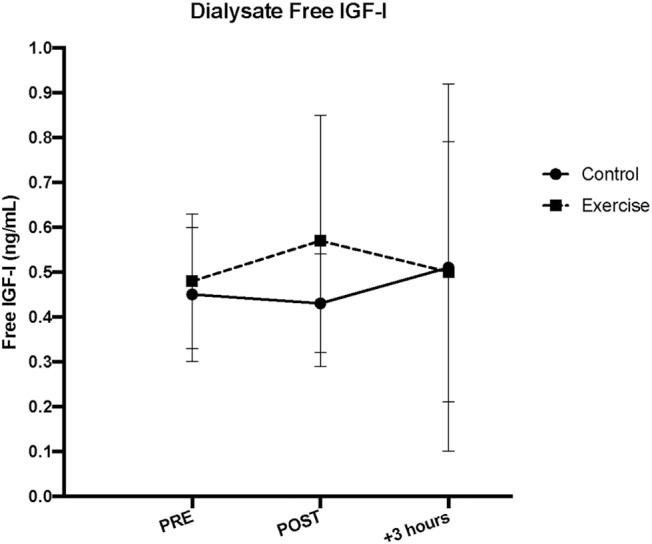
Interstitial fluid free insulin-like growth factor-I (IGF-I) concentrations measured via microdialysis dialysate in exercise vs. control leg pre, immediately post (IP), and 3 h post (+3 h) unilateral stretch shortening exercise.

**Figure 5:**
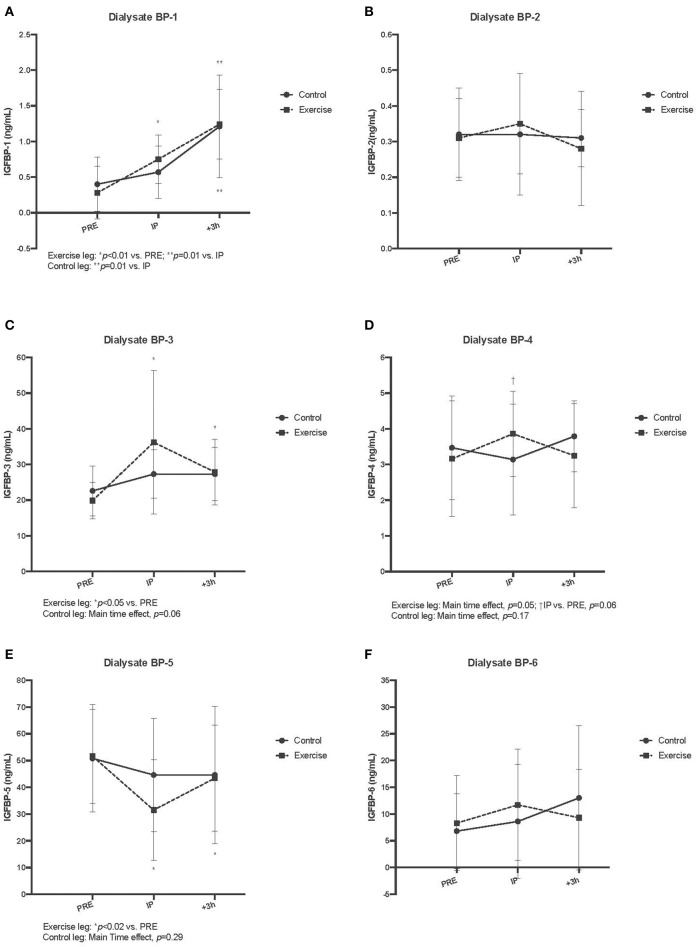
Interstitial fluid free insulin-like growth factor-I binding protein (IGFBP) concentrations measured via microdialysis dialysate in exercise vs. control for IGFBP-1 **(A)**, IGFBP-2 **(B)**, IGFBP-3 **(C)**, IGFBP-4 **(D)**, IGFBP-5 **(E)**, and IGFBP-6 **(F)** pre, immediately post (IP), 3 h post (+3 h), to unilateral stretch shortening exercise. *denotes statistical difference from pre-exercise (*p* < 0.05). Main and interaction effects are also noted in the figures.

Figure 5 displays the results for interstitial fluid IGFBPS. For dialysate BP-1, a significant main time effect was observed in both control and exercise leg musculature (*p* < 0.01 for both). Within the exercise leg, there was an increase throughout exercise and into recovery (Ex vs. Pre, *P* < 0.01; Rec vs. Ex, *p* = 0.01), whereas in the control leg, there was only a significant increase observed during the +3 h post recovery period (Rec vs. Ex, *p* = 0.01). Similar to serum BP-2, there were no acute changes in dialysate BP-2 with exercise (exercise leg, *p* = 0.14; control leg, *p* = 0.94). Dialysate BP-3 demonstrated an acute and sustained elevated concentration relative to baseline in the exercise leg (Ex and Rec vs. Pre, *p* < 0.05), whereas the control leg did not have a significant response (*p* = 0.06). For dialysate BP-4, there was a main time effect for the exercise leg only (*p* = 0.05), while the control leg did not change (*p* = 0.17). *Post-hoc* testing revealed that within the exercise leg, only the immediately post exercise time point was trending toward BP-4 being significantly increased (Ex vs. Pre, *p* = 0.06). Interestingly, there was a decrease in dialysate BP-5 in the exercise leg only, which remained below baseline values during the post recovery period (Ex and Rec vs. Pre, *p* < 0.02). Dialysate BP-5 did not change in the control leg (*p* = 0.29). Dialysate BP-6 did not change in either the exercise or control leg (*p* = 0.28 and 0.55, respectively) as a result of unilateral SSC exercise (Refer to Figure 4). Table 1 lists the comparison of IGF-I system component response patterns across the blood and muscle ISF for exercises and control leg during unilateral stretch shortening cycle exercise.

**Table 1:** Comparison of IGF-I system component response patterns during unilateral stretch shortening cycle exercise for blood (measured via veninpuncture) and exercised and control muscle interstitial fluid (ISF) (measured via microdialysis).

**IGF-I system component**	**Blood**	**Muscle ISF Exercise leg**	**Muscle ISF Control leg**
Total IGF-I	↑ (IP, +3 h post)	NA	NA
Free IGF-I	↔	↔	↔
IGFBP-1	↑ (+20 h post)	↑ (IP, +3 h post)	↑ (+3 h post)
IGFBP-2	↔	↔	↔
IGFBP-3	↑ (+20 h post)	↑ (IP, +3 h post)	↔
IGFBP-4	↑ (IP)	↑ (IP; *p* = 0.06)	↔
IGFBP-5	↔	↓ (IP, + 3 h post)	↔
IGFBP-6	↓ (+3 h post)	↔	↔

*↑, increase; ↓, decrease; ↔, no statistical significant change (p > 0.05); NA, not measured. This table was adapted from Figures 1–4*.

### EtOH O:I

We observed that microvascular blood flow was increased (decreased EtOH O:I ratio) during the exercise time period (O:I = 0.15 ± 0.07), which was different from both resting (O:I = 0.19 ± 0.06) and recovery (O:I = 0.20 ± 0.07) time points (*p* < 0.01 for both), but was not different between exercise (O:I = 0.18 ± 0.06) and control (O:I = 0.18 ± 0.06) leg musculature (main effect: *p* = 0.67).

### Interstitial Free IGF-I

Our estimated interstitial concentrations indicated that we recovered ~15% IGF-I through our microdialysis probe across both exercise and control leg musculature *in vivo*. Although the amount recovered was not different between exercise and control legs (*p* = 0.56), the recovery was slightly higher with exercise (15.82 ± 1.23%) over resting (15.16 ± 1.17%) and recovery (15.04 ± 1.27%) time points (*p* < 0.01 for both). Further, although the corrected interstitial IGF-I concentration was higher than both serum and dialysate concentrations as expected, the corrected interstitial free IGF-I muscle protein concentration demonstrated a similar pattern as the dialysate IGF-I concentration, such that there were no acute changes in either the exercise or control legs (main time effect, *p* = 0.77 and 0.66, respectively).

## Discussion

The experiment in the current study was designed to extend our knowledge of exercise influences on the IGF-I system by using microdialysis during unilateral stretch-shortening cycle leg exercise to measure IGF-I and its associated family of IGFBPs in muscle interstitial fluid (ISF). By sampling muscle ISF in a control vs. exercise leg, we were able to selectively delineate the effects of systemic vs. local effects on the IGF-I system in the extracellular space (i.e., interstitial fluid) surrounding contracted vs. non-contracted muscle. Novel and salient findings are that when compared to the control leg, the ISF of the exercised leg revealed localized and differential IGFBP responses. Specifically, exercised vs. control muscle ISF demonstrated increases in IGFBP-3 and IGFBP-4, and decreases in IGFBP-5 concentrations. We interpret these data and other emerging IGFBP data (1, 3, 12, 14, 28–30) to support our working hypothesis that a major influence of exercise on the IGF-I system across various biocompartments is via IGFBPs and perhaps even more so than alterations in total IGF-I circulating concentrations.

Exercise resulted in increased IGFBP-3 and IGFBP-4 and decreased IGFBP-5 muscle ISF concentrations. Given some of the known potentiating and inhibitory functions for IGFBPs, these findings could be generally considered favorable for IGF-I biological activity and subsequent muscle adaptation (1, 10, 31, 32). While IGFBP-3's main role in to modulate IGF-I bioavailability in the blood, it is interesting to note that IGFBP-4 and −5 are the most abundant IGFBPs in muscle (33). Comparing our results to those of the literature are difficult as we are aware of only two previous studies that measured IGFBPs in exercising muscle via microdialysis and reported no change in IGFBP-1 and IGFBP-3 proteolysis (21) and no change in IGFBP-3 and 4 (34). However, Olesen et al. (24) have measured IGFBPs via microdialysis in peritendinous connective tissue after running and reported increases in local IGFBP-4 concentrations. The current study is the first to concomitantly measure the entire IGFBP family (i.e., BPs 1-6) in both blood and muscle ISF, and thereby provides some important insight for exercise effects across these two important biocompartments. It is possible that the different temporal IGFBP concentration response patterns observed across these biocompartments may represent different physiological cascades impacting whole body metabolism and physiology vs. local muscle metabolic and recovery adaptations (35).

Under certain conditions, IGFBPs 4 and 5 have been shown to have both stimulatory and inhibitory actions (1, 16, 36, 37). Awede et al. (16) were the first to demonstrate regulation of IGFBP-4 and 5 gene expression by loading in mouse skeletal muscle. With overload, Awede et al. (16) demonstrated a 200% increase in IGFBP-4 mRNA levels and a 60% decrease in IGFBP-5 mRNA and a subsequent 200% increase with in IGFBP-5 mRNA with unloading, providing some of the earliest evidence implicating IGFBPs in adaptation to skeletal muscle loading. While Ewton et al. (31) have reported a dual role or IGFBP-5 in L6A1 myoblasts dependent on the culture medium, the underlying mechanisms for an inhibitory role reside during the early proliferative response of L6A1 cells to IGF-I by inhibition of: tyrosine phosphorylation of the cytoplasmic signaling molecules, IRS-1 and Shc, the activation of the MAP kinases, ERK1 and ERK2, the elevation in steady-state levels of the mRNA of the nuclear transcription factor c-fos, the early inhibition of elevation of myogenin mRNA, and increase in cell number (31). Other evidence also suggests an inhibitory role for IGFBP-5 such as comprised survival, growth, muscle development and fertility in mice (38), IGFBP-5 interaction with thrombospondin-1 to induce negative regulatory effects on IGF-I action (39), IGFBP-5 blocking of muscle differentiating IGF-I actions (40), and IGFBP-5 induced cell senescence (41). Thus, we interpret the decrease in ISF IGFBP-5 immediately post-exercise and +3 h into recovery in the exercise leg only to be a positive response with regard to muscle adaptation and conclude that this is also specific and localized within the ISF IGF-I system that has not been previously reported. The additional observation of ISF IGFBPs 3 and 4 changing before corresponding discernable changes in the circulation also substantiate the value of sampling and measuring the IGF-I system outside of the blood biocompartment to provide greater physiological insight and context (4, 7, 9, 18, 20, 21, 24, 34).

The exercise response for IGFBP-4 (*p* = 0.06 for the immediate post time point) was in the opposite direction (i.e., increased), less pronounced and not sustained when compared to IGFBP-5 response (decrease). While Miller et al. (34) did not report an IGFBP-4 increase after one-legged kicking exercise, Oleson et al. (24) reported an elevation in IGFBP-4 following exercise in peritendinous connective tissue ISF and noted that this increase preceded an elevation of procollagen type I carboxy-terminal propeptide (PICP). IGFBP-4 was also the only IGFBP to manifest an apparent increase at similar time points (immediate post exercise in both blood and ISF). While it is difficult to assign a definitive role for IGFBP-4 in muscle hypertrophy, a recent review by Clemmons suggests that given the available evidence in human studies, increased IGFBP-4 increases the supply of available IGF-I and if sufficient proteolytic activity is present this results in enhanced free IGF-I bioavailability and an anabolic response (1). In muscle ISF, IGFBP-3 was increased at the immediate post time point, similar to IGFBP-4. In the blood, IGFBP-3 was increased only at the +20 h post time point. Even though IGFBP-3 increases are considered as positive and stimulatory for circulating IGF-action and signaling, it is likely that similar conditions must exist for IGFBP-3 to be anabolic in muscle microenvironment (1, 10, 15).

While ISF IGFBPs 3, 4, and 5 demonstrated specific and localized exercise effects (changes were only observed in the exercised leg), IGFBP-1 increased in both the exercise and control leg likely reflecting an overall metabolic systemic effect (1, 12, 28, 32). In a study examining the effects of exercise mode and duration on 24-h IGF-I system recovery responses, IGFBP-1 was the only IGFBP that was sensitive to the exercise duration (IGFBP-1 was increased with longer duration exercise) (28). It was concluded that IGFBP-1 was a sensitive circulating biomarker reflecting the physiological strain by exercise. IGFBP-1 is typically considered inhibitory to IGF-I action and inversely proportional to insulin release (32). A recent report suggests that active recovery following heavy resistance exercise may attenuate circulating IGFBP-1 perhaps assisting in the facilitation of the recovery processes (30). Of note, circulating IGFBP-1 in the blood was increased for the +20 h post time point likely indicating the exercise was sufficiently metabolically taxing and recovery processes were still in effect the day after the exercise. When all blood and ISF IGFBP findings are considered, the 20+ h post time point was most coincident for IGFBP-1 in that elevations were observed in both blood and ISF. However, the temporal resolution pattern reveals that ISF IGFBP-1 increases post-exercise can be observed ~17 h before detected in the blood.

While the IGFBP family likely contributes to an amplified level of functional and regulatory diversity that serves to facilitate fine-tuning of IGF bioactivity and signaling (10), it is also recognized that IGFBPs possess IGF-independent actions (36). Of the major actions credited to IGFBPs (10): sequestration of IGF-1 away from the IGF-I receptor, promotion of IGF signaling by proteolytic cleavage and liberation of IGFs from IGF/IGBP complexes, trafficking and concentrating IGF-I toward receptor to provide availability and access, and IGF independent actions via binding to the IGFBP receptor, the independent actions of IGFBPs perhaps represent the most intriguing consideration within the context of our current findings. A number of characteristics of IGFBPs contribute to their amplified flexibility and versatility in influencing exercise-mediated adaptations (10): (1) distinct spatiotemporal expression patterns of IGFBP genes, (2) differences in ligand-binding affinity and selectivity, (3) different roles in the circulation including formation of binary and ternary complexes, (4) different abilities to interact with cell surface proteins, extracellular proteins, and other growth factors, (5) different subcellular localization, and (6) independent actions.

No significant changes were observed in interstitial fluid free IGF-I in either leg during the sampling period. This finding is consistent with previous reports also reporting no significant changes in ISF IGF-I following exercise (9, 20, 24). In our previous report indicating no change in ISF IGF-I after explosive, high-power exercise, we attempted to maximize the likelihood of detecting any potential change by using a larger molecular cutoff microdialysis probe (100 kDA) than previous studies had used (9). However, in our previous study, we removed the microdialysis catheters during exercise to safeguard against possible damage to the catheter integrity during muscle contractions. Removing the microdialysis catheter during exercise required a 45-min equilibration period post-exercise catheter insertion before ISF IGF-I could be sampled, therefore, we could not entirely dismiss the possibility that a bolus of IGF-I release was missed due to a diminished temporal resolution. Subsequent pilot work prior to the current study indicated that microdialysis catheter insertion during exercise was both viable and safe. Hence, any bolus of IGF-I release during exercise into the ISF should have been collected with the current experimental methodology. Circulating total, but not free, IGF-I was elevated at However, we cannot entirely dismiss the possibility that a rapid, small and transient ISF IGF-I change was missed due to microdialysis temporal resolution limitations. The immediate post and +3 h post and these results are consistent with some, but not all previous reports as the literature provides equivocal results for exercise and circulating IGF-I (refs). The lack of any measurable exercise-induced changes in IGF-I concentrations in muscle ISF among the current and other studies could suggest potentiated biological activity may reside in its partitioning among its family of binding proteins (7, 28, 35).

In summary, by measuring IGF-I and the IGFBP family in blood and muscle ISF via microdialysis after exercise, this study represents the most comprehensive characterization to date. Blood and ISF measures of the IGF-I system were fairly discordant implying that IGF-I measurement across biocompartments provides different information with regard to IGF-I action. By employing a unilateral stretch shortening cycle exercise, we were able to demonstrate differential and localized IGFBP responses in muscle ISF (i.e., increased IGFBP-3 and 4 accompanied by decreased IGFBP-5). We conclude that muscle contractions yield a local extracellular milieu whereby specific IGFBPs are altered. The physiological significance could be either through direct independent IGFBP actions or by influencing IGF-I bioactivity by sequestration or trafficking/delivery mechanisms. We also suggest that specific exercise-mediated IGF-I system influences might be better detected in ISF whereas blood measures may be more reflective of generalized whole body metabolic effects.

## Data Availability Statement

All datasets generated for this study are included in the article/supplementary material.

## Ethics Statement

The studies involving human participants were reviewed and approved by Ethics Committee of the Central Finnish Hospital District, Jyvaskyla IRB. The patients/participants provided their written informed consent to participate in this study.

## Author Contributions

BN, JA, ML, KH, and HK contributed to the experimental design. BN, JA, SG, RT, and HK contributed to data collection. All authors contributed to data analysis, writing, and interpretation.

## Conflict of Interest

The authors declare that the research was conducted in the absence of any commercial or financial relationships that could be construed as a potential conflict of interest.
